# Supplementary material for: Identification and functional analysis of non-coding regulatory small RNA FenSr3 in Bacillus amyloliquefaciens LPB-18
Source: PeerJ. 2023 May 15;11:e15236. doi: 10.7717/peerj.15236 (PMC10194069; doi:10.7717/peerj.15236)
Supplement: Supplemental Information 4 [file peerj-11-15236-s004.zip › KO/CK-vs-T1_map/map04540.html]

KEGG PATHWAY: Gap junction - Reference pathway


|  |  |
| --- | --- |
| **Gap junction - Reference pathway** |  |

[
Pathway menu
| Organism menu
| Pathway entry
| Show description
| User data mapping
]

Gap junctions contain intercellular channels that allow direct communication between the cytosolic compartments of adjacent cells. Each gap junction channel is formed by docking of two 'hemichannels', each containing six connexins, contributed by each neighboring cell. These channels permit the direct transfer of small molecules including ions, amino acids, nucleotides, second messengers and other metabolites between adjacent cells. Gap junctional communication is essential for many physiological events, including embryonic development, electrical coupling, metabolic transport, apoptosis, and tissue homeostasis. Communication through Gap Junction is sensitive to a variety of stimuli, including changes in the level of intracellular Ca2+, pH, transjunctional applied voltage and phosphorylation/dephosphorylation processes. This figure represents the possible activation routes of different protein kinases involved in Cx43 and Cx36 phosphorylation.

|  |  |  |
| --- | --- | --- |
| Reference pathway | 184% 150% 122% 100% 82% 67% 55% | 图片下载 |
